# Defensive coloration is not a reliable indicator of fungal infection in aposematic poison frogs

**DOI:** 10.1093/beheco/araf137

**Published:** 2025-11-20

**Authors:** Lia Schlippe Justicia, Carolin Dittrich, Ossi Nokelainen, Bibiana Rojas

**Affiliations:** Department of Interdisciplinary Life Sciences, Konrad Lorenz Institute of Ethology, University of Veterinary Medicine Vienna, Savoyenstraße 1, Vienna 1160, Austria; Department of Interdisciplinary Life Sciences, Konrad Lorenz Institute of Ethology, University of Veterinary Medicine Vienna, Savoyenstraße 1, Vienna 1160, Austria; Institute of Hydrobiology, Technische Universität Dresden, Zellescher Weg 40, Dresden 01217, Germany; Department of Biology and Environmental Science, University of Jyväskylä, P.O. Box 35, Jyväskylä 40014, Finland; Department of Biology and Environmental Science, University of Jyväskylä, P.O. Box 35, Jyväskylä 40014, Finland; Open Science Centre, University of Jyväskylä, P.O. Box 35, Jyväskylä 40014, Finland; Department of Interdisciplinary Life Sciences, Konrad Lorenz Institute of Ethology, University of Veterinary Medicine Vienna, Savoyenstraße 1, Vienna 1160, Austria

**Keywords:** Amazon, carotenoids, chytrid fungus, *Dendrobates tinctorius*, melanin, pathogens, poison frog, visual signal

## Abstract

The expression of visual signals such as coloration can be altered by parasitic or pathogenic infections through multiple pathways, including resource reallocation, impaired tissue structure, and reduced pigment acquisition. These effects may compromise the functions of coloration and overall fitness. Conversely, the link between pigments and immunological defences can aid differently colored individuals in coping with infection. While the pigmentation-condition association has been widely studied in the context of sexual selection, far less is known about how pathogens affect defensive coloration, such as aposematic signals. Here, we investigated whether infection by the fungal pathogen *Batrachochytrium dendrobatidis* (*Bd*) is reflected in characteristics of the melanin- and/or carotenoid-based coloration of the aposematic poison frog *Dendrobates tinctorius* in the wild. Using ddPCR to identify the frogs' infection status, and multispectral digital imaging to quantify their coloration traits, we show that neither type of coloration is a reliable indicator of *Bd* infection. Instead, body size influenced both infection outcomes and coloration, with sex-specific patterns suggesting potential ontogenetic or life-history trade-offs. Our findings highlight that the links between color expression and condition are more context- and taxa-dependent than often assumed, and suggest that, in *D. tinctorius*, defensive signals may remain stable despite pathogen exposure.

## Teaser

Bright warning colors in animals often signal danger to predators and are thought to reflect health. But do these colors change when individuals are infected? We studied wild poison frogs infected by a common skin fungus and found that their striking colors remained stable. Instead, body size was linked to color differences and infection risk. Our findings suggest that the reliability of color as a signal of condition depends on context and may not apply broadly across species.

## Introduction

Coloration in animals plays a central role in a variety of ecological and evolutionary processes including predator avoidance and intraspecific communication. For example, color patterns can play a defensive role against predators via crypsis or aposematism (Cott 1940). While cryptic coloration reduces the likelihood of detection or recognition by predators (Endler 1988; Nokelainen and Stevens 2016), aposematic signals rely on conspicuous color patterns coupled with secondary defences, such as chemical compounds, to deter attacks by advertising unpalatability or toxicity to potential predators (Poulton 1890; Rojas et al. 2015). Additionally, coloration can play a key role in sexual selection, as bright colors and elaborate ornamental displays are often used as visual signals in agonistic encounters (Pryke and Griffith 2006) and mate choice (Hill 1991). Because the production and maintenance of color traits is energetically costly, these traits are, in many cases, honest signals of individuals' fitness and parental care abilities in a wide range of species (Hill 1991; Weaver et al. 2017).

In vertebrates, coloration is primarily determined by the type, concentration, and spatial arrangement of specific pigments within the skin or integument. Bright colors such as red and yellow are often produced by carotenoid and pteridine pigments, while darker tones typically result from melanin, particularly eumelanin (Fitzpatrick 1974; Hill and McGraw 2006). In addition, structural components can produce blue or iridescent colors by reflecting and scattering light, often in combination with underlying pigments (Sun et al. 2013). Importantly, these pigments are obtained through different pathways: while pteridines and melanin are produced *de novo* (Hearing 1993; D’Alba and Shawkey 2019), carotenoids cannot be synthesized by vertebrates but have to be acquired through diet (Goodwin 2012). As a result, carotenoid-based coloration is closely linked to the physiological condition and foraging abilities of individuals (Brush and Power 1976; Stückler et al. 2022), whereas melanin-based coloration is typically less dependent on environmental inputs and more strongly regulated by genetic factors, although some ecological factors can still modulate its expression (Roulin 2016).

In addition to their role in coloration, pigments serve important physiological functions, particularly in immune defence. For example, carotenoids act as antioxidants and contribute to a range of immune processes, influencing resistance to parasites (Alonso-Alvarez et al. 2004). Similarly, the physical-chemical properties of melanin suggest antimicrobial activity (Mackintosh 2001), as its codifying genes have pleiotropic effects on melanocortin receptors that regulate immune responses (Ducrest et al. 2008; Gangoso et al. 2011; Côte et al. 2018). Beyond vertebrates, melanin also plays a central role in insect immunity, where it is used to encapsulate and kill invading pathogens (Hillyer 2016). Therefore, pigmentation patterns, particularly those based on carotenoids and melanin, can serve as valuable indicators of both external ecological factors and internal physiological state.

Because of the physiological costs associated with pigment production and maintenance, coloration is susceptible to disruption by parasites and pathogens. Infected individuals may experience trade-offs in resource allocation between the immune system, which demands substantial energy upon infection, and other fitness-related traits, including ornamentation and signal expression (Sheldon and Verhulst 1996). This resource trade-off can result, among other effects, in alterations in color intensity, shifts in dominant wavelength, and/or loss of brightness, reducing the quality of ornaments and body coloration (Milinski and Bakker 1990; Hill and Brawner 1998; Rodrigo et al. 2016; Penha et al. 2020). While such effects have been extensively documented in birds and fish, particularly in the context of sexual selection (eg, Houde and Torio 1992; Mougeot et al. 2010; Penha et al. 2020), they have received comparatively little attention in amphibians (but see Pröhl et al. 2013; Kindermann et al. 2017; Longo et al. 2020; Auld 2024) and in the context of anti-predator coloration. As a group exhibiting functionally diverse coloration (Rudh and Qvarnström 2013; Rojas 2017; Rojas et al. 2023) and commonly infected with the widespread chytrid fungus *Batrachochytrium dendrobatidis* (hereafter *Bd*), amphibians are excellent models for studying infection-driven changes on color traits.

*Bd* infects amphibians' keratinized tissues, causing chytridiomycosis, an infectious disease that disrupts critical functions such as skin osmoregulation (Voyles et al. 2009) and immune responses (Fites et al. 2013), often leading to mortality (Voyles et al. 2009). Pathological manifestations of *Bd* infection include hyperkeratosis, inflammation, and increased rates of skin sloughing (Berger et al. 2005; Ohmer et al. 2015, 2017), which could directly alter the optical properties of skin and thus influence color expression. Indirectly, *Bd* infection may constrain pigment acquisition and/or production by diverting vital resources towards immune responses, and by inducing secondary costs such as reduced feeding rates (Peterson et al. 2013) and heightened metabolic expenditure (McQuigg et al. 2023).

One of the most color-diverse and well-studied amphibian groups is dendrobatid poison frogs (Stynoski et al. 2015). Multiple species in this group present conspicuous color patterns (Rojas 2017) paired with alkaloid defences (Daly et al. 2005), signaling unprofitability to potential predators such as birds (Saporito et al. 2007; Maan and Cummings 2012; Rojas et al. 2014; Lawrence et al. 2019)—offering a classic example of aposematism (Poulton 1890). This defensive coloration is produced by a combination of pigment types, including diet-derived carotenoids, endogenously synthesized pteridines, and eumelanins (Twomey et al. 2020). As these pigment classes originate through distinct physiological pathways, their expression may respond differently to infection.

Here, we investigated whether infection with *Bd* alters the dorsal coloration of the dyeing poison frog *Dendrobates tinctorius* and, if so, whether melanin- and carotenoid-based coloration is impacted differently. Additionally, we examined whether *Bd* infection prevalence and intensity were explained by sex, body size and body condition. We predicted that:

1. If coloration reflects *Bd* infection status, infected frogs will show duller, less saturated coloration due to trade-offs between immune response and pigment expression.
2. *Bd* infection will more strongly affect carotenoid-based coloration, given its dietary origin and potential infection-related reductions in foraging or metabolic efficiency. Alternatively, as melanin is synthesized endogenously, we could expect its expression to be suppressed due to physiological costs, or upregulated as a physiological response to stress or infection, which can stimulate melanin production through hormonal pathways.
3. Individuals in poorer body condition will be more likely infected and carry higher *Bd* loads, likely due to either compromised immune system or to infection-driven declines in condition.

## Material and methods

### Study species

**Fig. 1.**
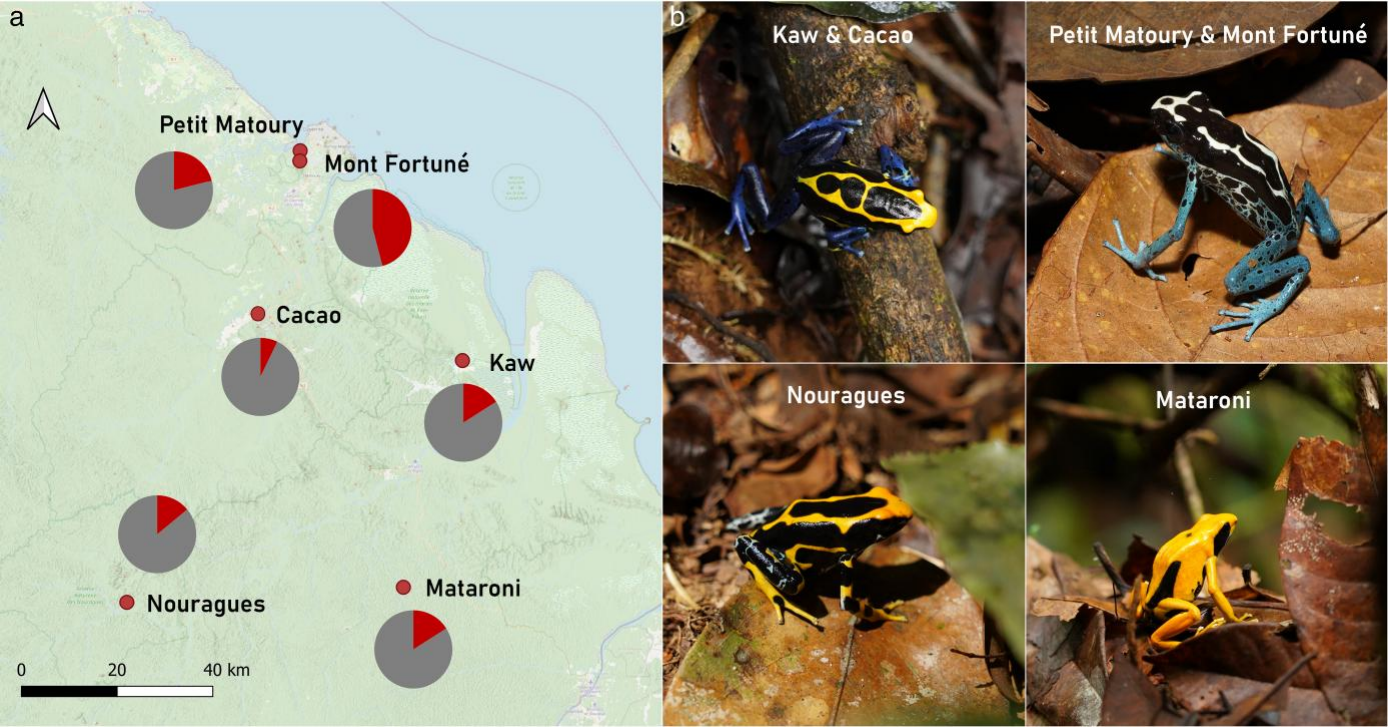
(a) Map showing the location of the six studied populations (red dots) and the prevalence of *Bd* infection at each site (pie chart: red corresponds to infected and gray to uninfected frogs). (b) Inter-population color variation in *Dendrobates tinctorius* at our study sites. Note that one site was excluded due to low sample size and, thus, not represented in the figure. Photos: Martin Mayer.

*Dendrobates tinctorius* is a diurnal species endemic to the eastern Guiana Shield (Rojas and Pašukonis 2019), at elevations between 0 and 600 m.a.s.l. (Noonan & Gaucher 2006). Individuals of both sexes exhibit conspicuous and highly variable color patterns within (Rojas and Endler 2013) and between (Wollenberg et al. 2008; Mayer et al. 2025) populations (Fig. 1). In most cases, these color patterns consist of light-colored (eg, yellow, white) markings of different shapes and sizes on a black background (Wollenberg et al. 2008). These conspicuous color patterns are paired with alkaloid-based chemical defences (Daly et al. 2005) which makes these frogs unpalatable for avian predators (Lawrence et al. 2019, 2023). Size is a highly variable trait among populations, with a snout-vent length (SVL) ranging between 2.8 and 5.8 cm in females, and between 3.0 and 4.9 cm in males (Schlippe Justicia et al. 2024; this study). Males provide their offspring with elaborate parental care, which includes clutch attendance and tadpole transportation to small water bodies within plant structures (ie phytotelmata) (Rojas 2014; Rojas and Pašukonis 2019).

### Frog sampling

From January to March 2023 we sampled six populations of *Dendrobates tinctorius* across French Guiana, where *Bd* was first documented in 2012 with higher prevalence in dendrobatids than in other families (Courtois et al. 2012). Frogs were captured by hand, using new nitrile gloves for each individual, and swabbed with a sterile nylon-flocked swab (4N6FLOQSwabs, ThermoFisher Scientific) by rotating it five times over the dorsum, venter, thighs, and each side of the body. Swabs were immediately stored individually in DNA/RNA-shield (Zymo Research Corp, Irvine, CA) to avoid DNA degradation after field collection. A maximum of 50 individuals was swabbed at each study site.

Every frog was subsequently weighed to the nearest 0.01 g and sexed based on the width of their toe discs in relation to body size, which is larger in males than in females (Rojas and Endler 2013). We photographed the ventral, dorsal and lateral patterns of each frog against graph paper for (1) individual identification, (2) measurement of snout–vent length (SVL), and (3) quantitative color analysis. All individuals were released right after at the point of capture. Boots and field equipment were brushed and disinfected with ethanol between locations to minimize the risk of spreading *Bd*.

### DNA extraction and ddPCR

DNA was extracted from swabs using the Quick DNA Magbead Plus Kit (Zymo Research, Irvine, CA) following the manufacturer's protocol for microbial samples. For each round of extractions, we processed a blank sample (DNA/RNA-shield only) as a negative control.

The DNA extracts were processed and read on a Bio-Rad QX200 Droplet Digital PCR (ddPCR) system to quantify *Bd* and *D. tinctorius* DNA, targeting the ITS region and the RAG1 gene respectively. Each sample contained 10 µL ddPCR Supermix for probes (No dUTP, Bio-Rad Laboratories, Inc.), 1.8 µL of species-specific primer (0.9 µL forward and reverse primer each, 20 µM), 0.5 µL of targets-specific probe (10 µM), 0.4 µl H_2_O, and 5 µL of template DNA, in a total volume of 20 µL. The primer and probe sequences can be found in the Supplementary material (S1). Endpoint PCR amplification was conducted on a Biometra Thermal Cycler (Biometra TOne Series) under the following cycling conditions: Activation at 95 °C for 10 min, followed by 50 cycles of amplification at 94 °C for 30 s (denaturation) and 60 °C for 2 min (annealing), followed by droplet stabilization at 98 °C for 10 min and a final infinite hold at 4 °C (ramp rate 0.1 °C/sec). In each run, we included a nontemplate control (H_2_O), one negative extraction control and a positive control (mix of *D. tinctorius* and *Bd* DNA, 3.5 µL and 1.5 µL, respectively).

QX Manager 1.2 Software (Bio-Rad Laboratories, Inc.) was used to analyse positive and negative droplets, according to the manufacturer's instructions. Samples with an average number of accepted droplets under 10,000 were considered nonquantifiable for analysis and were rerun. For each run, we set a threshold for *Bd* detection based on the positive and negative control, and samples that contained a low number of positive droplets (<10) were run twice. In addition, in order to minimize false positives, we only considered a sample as *Bd* positive if it had a minimum of 3 positive droplets in the two runs (Dobnik et al. 2015). We averaged *Bd* loads between duplicates. We defined *Bd* prevalence as the number of infected frogs out of the total number of frogs sampled, and *Bd* load (also referred to as infection intensity) as the number of *Bd* DNA copies detected per μL of each swab sample.

### Color measurements

We photographed frogs under natural light conditions using a Sony Alpha 7 III camera with 24 to 105 mm lens, next to a ColorChecker Passport (X-Rite, Michigan, USA) and a scale. For each individual, we analysed one picture of the dorsal pattern in RAW format using the Multispectral Image Calibration and Analysis (MICA) Toolbox add-on version 2.2.2 (Troscianko and Stevens 2015) in the software ImageJ (Abramoff et al. 2004). Images were calibrated using the 24-tile color checker placed within each photo to correct for variation in light conditions among photographs. As this species exhibits a very dark black pigmentation, each image was calibrated twice to deal with the distinct reflectance properties of the yellow (carotenoid-based) and black (melanin-based) skin coloration of *D. tinctorius*. Using the conventional calibration (with black and white point reflectances) led to negative values in the black regions, an artifact of the calibration process that would misrepresent the true coloration. Thus, to accurately quantify the black areas, we calibrated the images using a custom black reference derived from confirmed spectrometer measurements (obtained during the course of a previous study; B. Rojas, unpublished data) of 24 wild individuals' black patches. This approach allowed us to adjust the black tile value in a biologically meaningful way. For the yellow coloration, we retained the standard calibration using the black, neutral 3, and white tiles of the color checker, as these values adequately captured the reflectance range. While using two separate calibrations impedes direct comparison between the yellow and black patches, our approach ensured accurate within-color quantification of reflectance for both pigmentation types.

Following calibration, we selected five regions of interest (ROIs) in yellow patches and three in black patches for measurement. ROIs were placed on body areas consistently exhibiting the same color type (eg, yellow in snout, black in the laterals of the dorsal pelvic) across individuals, and were manually selected to avoid visual artifacts such as reflections or shade. Calibrated images were then converted into multispectral image files using standard D65 illumination as the reference light source. From these, we extracted reflectance values for each ROI in the RGB color space, corresponding to the visible spectrum: blue (∼400 to 500 nm), green (∼500 to 600 nm), and red (∼600 to 700 nm). RGB values were then converted to the device-independent CIELAB color space, providing a more perceptually uniform space (Stevens et al. 2007), and multiple color parameters were calculated independent of any visual system.

For the yellow coloration analysis, we measured: hue (the dominant wavelength or color type), saturation (the intensity or richness of color compared with white light), and brightness (the total amount of light reflected), whereas the differences in black coloration were investigated using brightness only, as variations in black are primarily due to differences in light reflectance, not color intensity or type (Endler 1990). ROIs measurements were averaged separately for yellow and black regions to obtain one mean value per color per individual. We retained a single dorsal photograph per individual for the color analysis, removing juvenile and poor-quality pictures, for a final sample size of 201 images.

### Statistical analyses

Due to low sample size (*N* = 14), we excluded individuals from one population and juvenile frog observations from our analysis, keeping only male and female adult frogs from five populations. Data regarding juveniles and individuals from the excluded population are thus kept descriptive in the results section. Frog body condition was determined for each individual using a “scaled mass index” (SMI), following Peig and Green's (2009, 2010) formula, using the mean SVL of the population as L0 value. SMI was calculated separately for each population to account for population-specific allometric relationships. Similarly, prior to statistical analyses, both SMI and SVL were scaled separately per site to account for consistent differences in average body size across populations (Schlippe Justicia et al. 2024).

To determine whether *Bd* prevalence and infection intensity were explained by frog traits, we used two regression approaches. For *Bd* prevalence, we used a generalized linear mixed model (GLMM) with a binomial distribution and infection status (*Bd+*, *Bd*−) as the response variable. For infection intensity, we used a linear model with a Gaussian distribution and log-transformed *Bd* loads of infected individuals as the response variable. In both models, we included sex, body condition, scaled SVL, and the interaction between sex and SVL (to account for sexual size dimorphism) as fixed effects. Population was modeled as a random intercept in the prevalence model to account for potential nonindependence within study sites and background variation across locations. For the intensity model, population was included as a fixed effect due to model singularity when specified as random effect, caused by minimal between-population variance or model overfitting.

To investigate whether frog dorsal coloration is influenced by *Bd* infection, we ran regression models on data combined across all populations. Color parameters were modeled separately. For yellow coloration analysis, brightness and saturation were used as response variables in linear regression models, while hue was analysed using a Bayesian circular regression model fitted with the R package "bpnreg" (Cremers 2021; version 2.0.3) to account for the circular nature of this variable. For the analysis of black coloration, we modeled brightness only, using a linear regression. Response variables were log-transformed when necessary to achieve normality of residuals. In addition to *Bd* infection status (infected or noninfected), fixed effects included sex, body condition and SVL scaled per site, population, and the interactions between (1) sex and scaled SVL, (2) population and infection status (to account for differences in *Bd* prevalence among populations), and (3) body condition and *Bd* infection status (to explore whether the effect of infection on coloration depended on the frogs' condition). The phenotypic variables were included as they can also influence frog coloration (Brenes-Soto et al. 2017; Stephenson and Christensen 2023). To assess whether infection intensity (ie, *Bd* load) altered coloration, we ran models restricted to infected individuals, with *Bd* load as a predictor and the same model structure as above.

Body mass was highly correlated with frog SVL and was therefore excluded from the models. None of the other variables were highly correlated (all Pearson correlation coefficients <0.4). All numeric fixed effects were scaled and centered to avoid convergence issues and to allow comparisons of relative effect sizes. We fitted GLMMs using the package lme4 (Bates et al., 2015; version 1.1.34). Model simplification was performed through backward selection based on Akaike's Information Criterion corrected for small sample size (AICc) (Burnham et al. 2011), implemented via the R package “MuMIn” (Barton 2020; version 1.47.5), but full model outputs are provided in the Supplementary material (Table S2). To evaluate the contribution of predictors in the final model, we considered parameters uninformative if their 95% confidence intervals (CI) include zero (Arnold 2010), focusing on effect sizes and model support rather than statistical significance. *P*-values were only used in post hoc pairwise comparisons, where *P* < 0.05 was considered statistically significant and *P* values between 0.05 and 0.1 were considered a trend. Model fit and assumptions were verified by examining residuals versus fitted values (Zuur and Ieno 2016) and through diagnostic tests for dispersion and deviation using the R package “DHARMa” (Hartig 2022; version 0.4.7). All statistical analyses were conducted in R (R Core Team 2024; version 4.4.2).

## Results

### Patterns of *Bd* infection prevalence and intensity

We collected a total of 239 skin swabs (135 females, 89 males, 15 juveniles) from the six sampled populations of *D. tinctorius* to determine their infection status. Overall *Bd* infection prevalence was 21% for adult frogs, while only two juveniles (13%) were infected with loads of 41.2 and 387.6 *Bd* copies/μL. Infected adult frogs were detected in all study sites with prevalence ranging from 8% to 46% (Fig. 1) and *Bd* loads between 0.2 and 2027 copies/μL (mean ± SD: 173 ± 411.2) (Table 1).

**Table 1.** For each of the study areas, overview of the total number of adult dyeing poison frogs (*Dendrobates tinctorius*) sampled, the percentage of frogs infected with *Batrachochytrium dendrobatidis* (prevalence), and the mean *Bd* load and total range.

Study site	Sex	Adult frogs	*Bd* prevalence	Mean *Bd* load (range)
Nouragues	Male	21	19.0	59.8 (1.4 to 192.5)
	Female	28	10.7	172.3 (0.2 to 417.3)
Mataroni	Male	15	6.7	49.4
	Female	33	21.2	41.9 (0.3 to 243.6)
Kaw	Male	22	9.1	83.9 (0.2 to 167.7)
	Female	24	20.8	5.9 (0.4 to 23.4)
Mont Fortuné	Male	12	41.7	5.8 (0.3 to 20.1)
	Female	23	47.8	193.9 (0.3 to 1451.7)
Petit Matoury	Male	13	23.1	823.6 (1.13 to 2027.1)
	Female	21	23.8	389.4 (0.4 to 1376.8)
Cacao*	Male	6	0	…
	Female	6	16.7	1.1

Values are shown separately for males and females. Cacao*, with only one infected adult, was excluded from the main analysis due to low sample size.

**Fig. 2.**
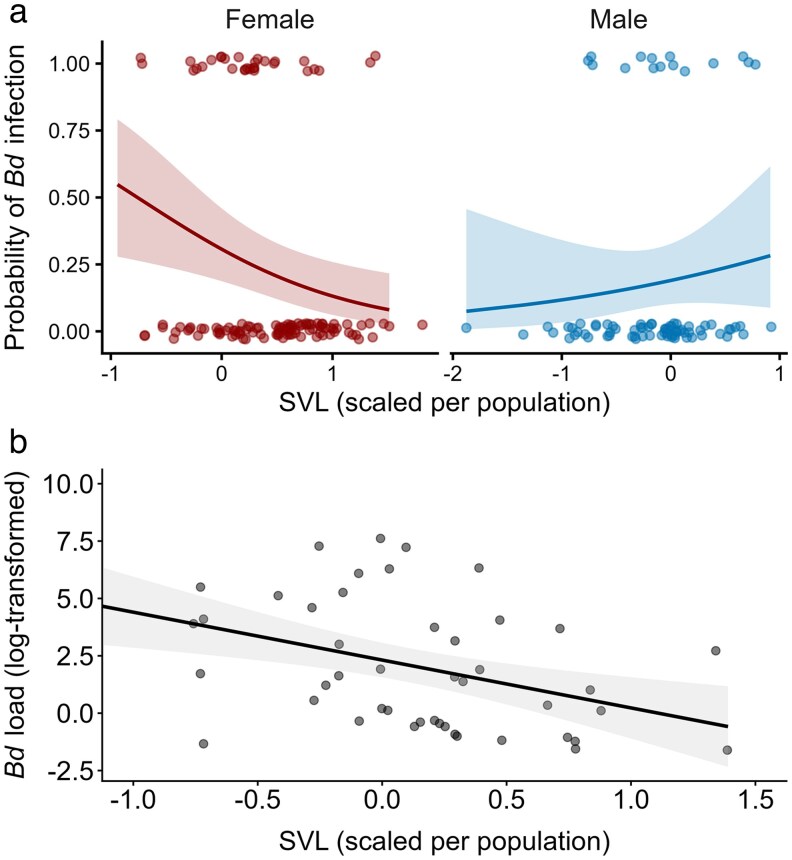
Predicted effect of population-scaled SVL on (a) the probability of *Bd* infection by sex, showing an informative effect only for females, and (b) log-transformed infection intensity (ie *Bd* load). Lines represent model predictions with 95% CI; raw data points are shown as dots. One data point (SVL = −2.25) was excluded for visualization purposes, but included in the models.

The probability of being infected decreased with increasing SVL in females, but not in males (Table 2a, Fig. 2a). Moreover, there was a trend suggesting that females were more likely to be infected than males (23.7% vs 16.8% overall), but the 95% confidence interval overlapped zero, indicating considerable uncertainty in the estimate. Body condition was not retained in the best model (Table 2a). In contrast, the infection intensity model showed a negative association between *Bd* load and SVL regardless of sex, meaning that larger individuals carried lower infection loads (Fig. 2b). Body condition was also retained in the best model as a weak trend, suggesting a negative effect of *Bd* load on body condition. No other variables were retained in the final model (Table 2b).

**Table 2.** Estimates, standard errors (SE), lower (LCI) and upper (UCI) 95% confidence intervals of the variables associated with (a) the probability of infection for 212 *Dendrobates tinctorius* adults from five populations, and (b) *Bd* load of 46 infected adult frogs.

*(a) Bd infection status*				
Parameter	Estimate	SE	LCI	UCI
**Intercept**	**−0**.**82**	**0**.**33**	**−1**.**58**	**−0**.**09**
**SVL**	**−1**.**07**	**0**.**40**	**−1**.**92**	**−0**.**32**
Sex (Male)	−0.64	0.39	−1.41	0.11
SVL*Sex (Male)	**1**.**64**	**0**.**74**	**0**.**22**	**3**.**17**

*(b) Bd infection load*				
Parameter	Estimate	SE	LCI	UCI
**Intercept**	**2**.**30**	**0**.**37**	**1**.**54**	**3**.**05**
**SVL**	**−2**.**09**	**0**.**61**	**−3**.**32**	**−0**.**85**
SMI	−0.63	0.31	−1.26	<0.01

Informative parameters are shown in bold (95% confidence intervals do not overlap zero).

### Melanin-based coloration

**Fig. 3.**
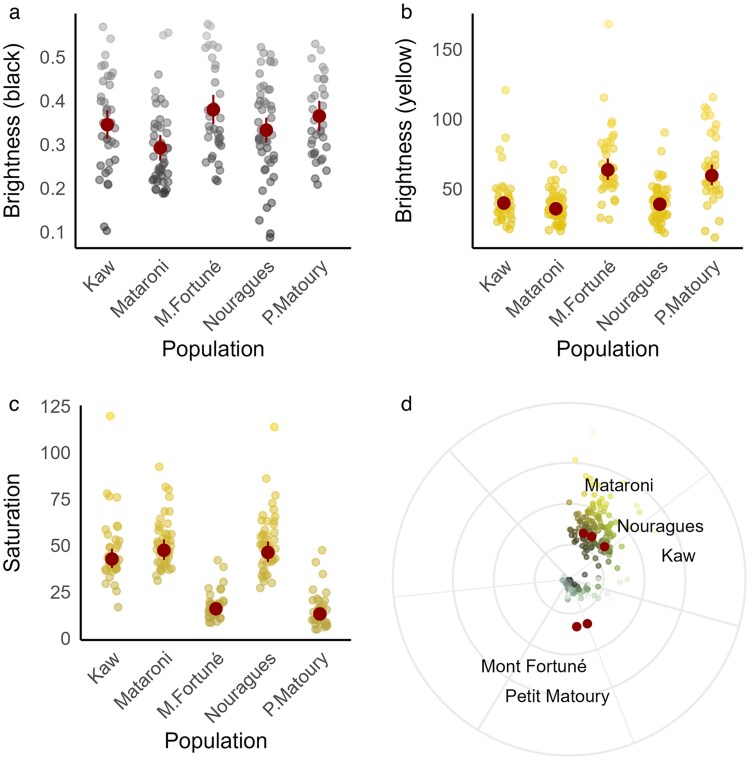
Mean values and 95% CI (red dots and bars) for each population: (a) brightness of the dorsal black coloration, and (b) brightness, (c) saturation, and (d) hue of the dorsal yellow coloration. Raw data points are overlaid as colored dots to illustrate each color parameter (note: dot colors are for visualization only and do not reflect actual color values). In all plots, frogs from Petit Matoury and Mont Fortuné show significantly different values compared with the other populations.

We found no evidence that *Bd* infection is associated with black dorsal coloration. In our analysis, population was the only predictor retained after model selection explaining variation in brightness (Table S3a). Post-hoc pairwise comparisons revealed that individuals from Mataroni had significantly lower brightness values compared with those from Petit Matoury and Mont Fortuné, while no significant differences were found among the other populations (Table S4a; Fig. 3a). When including *Bd* loads (ie analysis for infected individuals only), sex and body condition were retained in the best-supported model, although they were statistically uninformative (sex: estimate ± SE = 0.05 ± 0.03, 95% CI = −0.01 to 0.11; body condition: estimate ± SE = 0.02 ± 0.01, 95% CI = −0.01 to 0.05). No other variables or interaction terms were retained (Table S3b).

### Carotenoid-based coloration

When analysing the yellow dorsal coloration, we found a strong effect of population on brightness, saturation and hue, consistent with observed differences in morphotypes across populations (Table 3; Fig. 3b–d). In all three color metrics, frogs from Petit Matoury and Mont Fortuné significantly differed from all other populations, as confirmed by pairwise comparisons (Table S4b–d).

**Table 3.** Estimates and standard errors (SE) from linear models for the brightness and saturation of the yellow coloration.

Parameter	Brightness estimate (±SE)	Saturation estimate (±SE)	Hue effect (95% CI)
*(a) Analysis including *Bd* infection status*			
Intercept	**3.68** ± **0.06**	**3.74** ± **0.07**	**72.5** (**70.2 to 74.9)**
Sex (Male)	**…**	0.02 ± 0.06	5.0 (−151.8 to 149.4)
SVL	**…**	**0.14** ± **0.06**	34.4 (−30.5 to 64.2)
Population (Mataroni)	−0.11 ± 0.08	0.10 ± 0.08	29.6 (−8.8 to 46.0)
Population (M. Fortuné)	**0.47** ± **0.09**	**−0.99** **±** **0.09**	**−125.3** (**−134.5 to −118.4)**
Population (Nouragues)	**−**0.02 ± 0.08	0.08 ± 0.08	9.1 (−74.2 to 49.3)
Population (P. Matoury)	**0.40** ± **0.09**	**−1.18** **±** **0.09**	**−122.8** (**−130.7 to −116.9)**
SVL:Sex (Male)	…	**−0.26** **±** **0.10**	−120.8 (−168.9 to 158.7)
*(b) Analysis including Bd load*			
Intercept	**3.73** ± **0.14**	**3.70** ± **0.17**	**70.7** (**66.9 to 74.5)**
*Bd* load	−0.04 ± 0.02	…	68.3 (−95.5 to 146.3)
Sex (Male)	…	0.24 ± 0.14	−9.7 (−154.4 to 152.5**)**
Population (Mataroni)	−0.10 ± 0.19	0.22 ± 0.22	−23.8 (−96.9 to 46.3)
Population (M. Fortuné)	**0.51** ± **0.17**	**−0.93** **±** **0.20**	**−121.9** (**−136.3 to −114.2)**
Population (Nouragues)	0.04 ± 0.20	0.04 ± 0.23	−22.7 (−106.1 to 60.4)
Population (P. Matoury)	**0.49** ± **0.20**	**−1.19** **±** **0.22**	−**117.3** (**−127.2 to −111.2)**

Estimates for hue are derived from Bayesian circular regression and are shown as posterior mean hue direction (in degrees) with 95% credible intervals. Results are shown for models including (a) *Bd* infection status (all individuals) and (b) *Bd* load (infected individuals only) as predictors. Informative parameters (ie, 95% confidence or credible intervals not overlapping zero) are highlighted in bold, and empty fields indicate that the specific parameters were not retained in the best model.

**Fig. 4.**
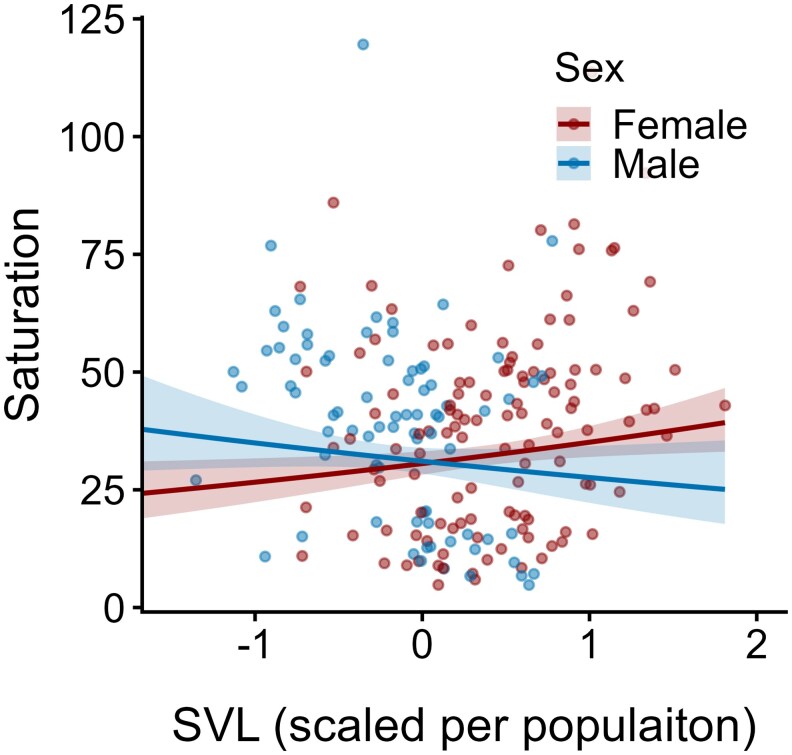
Predicted effect of population-scaled SVL on yellow coloration saturation, shown separately by sex. Lines represent model predictions with 95% CI; raw data points are overlaid as dots. One extreme data point (SVL = −2.25) was excluded from the plot for visualization clarity, but retained in the statistical analysis.

For saturation, SVL was also informative, with its effect dependent on sex. While females showed an increase in saturation with body size (ie bigger females have more saturated coloration), the opposite association was observed in males (Table 3a; Fig. 4). Similarly, the circular regression revealed a sex-specific relationship between hue and SVL, indicating a potential interaction between the two variables. In females, hue shifted towards “warmer” tones with increasing body size, whereas in males it shifted in the opposite direction. However, the overlapping credible intervals indicate considerable uncertainty in the estimated effects (Table 3a). Infection status, sex, body condition, and the interaction between site and infection status did not contribute meaningfully to explaining variation in yellow coloration.

When analysing infected individuals only, population remained the strongest predictor of variation across all three color metrics (Table 3b). In addition, brightness tended to decrease with increasing *Bd* load, but the effect was statistically uninformative (estimate ± SE = −0.04 ± 0.02, *P* = 0.06, 95% CI overlaps zero). Similarly, males tended to exhibit higher saturation than females, but this effect was also uninformative (estimate ± SE = 0.24 ± 0.14, *P* = 0.09, 95% CI overlaps zero).

## Discussion

We investigated whether dorsal coloration in wild *Dendrobates tinctorius* adults reflects infection status with *Batrachochytrium dendrobatidis* (*Bd*), a major driver of amphibian population declines worldwide (Houlahan et al. 2000; Fisher and Garner 2020). Contrary to our expectations, we found no evidence correlating *Bd* infection with either carotenoid- or melanin-based coloration in this species. These results suggest that, at least in our study populations and under current infection conditions, the expression of dorsal color traits is not a reliable indicator of *Bd* infection. This could indicate either an absence of trade-offs between infection-induced energy demands and pigment allocation, or that, under persistent exposure to *Bd*, selection has favored individuals capable of minimizing the physiological costs of infection on coloration. In line with the handicap principle, this pattern may reflect a long-term equilibrium in which only individuals able to sustain costly signals under permanent pathogen pressure persist, leaving little room for color variation despite infection. In either case, our findings highlight the potential resilience of wild *D. tinctorius* populations to *Bd* exposure. As melanin and carotenoid pigments differ in their physiological functions and metabolic origins, we analysed their potential relationships with infection status and load separately, considering both immune and signaling roles.

### Melanin-based coloration

Given that *Bd* infection can alter skin structure and integrity in amphibians, we expected it might also influence melanin-based coloration in *D. tinctorius*. Previous studies have reported infection-related changes such as hyperkeratosis, hyperplasia, or increased skin sloughing (Berger et al. 2005; Ohmer et al. 2015, 2017), all of which could affect the interaction between light and underlying chromatophores. If *Bd*-induced damage altered skin translucency or disrupted melanophore layers, we might expect increased brightness in infected frogs. Yet, our results did not support this hypothesis.

Melanin-based coloration is also closely linked to immune function (Côte et al. 2018). Melanin molecules possess toxic properties that can inhibit pathogen proliferation (Mackintosh 2001), and melanocortins may modulate immune activity by binding to receptors in both skin and immune cells (Gangoso et al. 2011). For instance, darker melanic pigeons (*Columba livia*) had lower parasite intensities and stronger immune responses than lighter individuals (Jacquin et al. 2011). This raises the possibility that baseline melanin levels could be shaping the frogs' ability to mount a response to *Bd* infection. However, this association may not be universal. In a laboratory experiment, Venesky et al. (2015) found that in the red-backed salamander (*Plethodon cinereus*), the darker, more melanized morph, exhibited higher *Bd* infection prevalence and mortality than the lighter morph. This unexpected result suggests that other factors, such as differential behavior or physiological stress, may override the protective role of melanin in some amphibians. In our study, the link between melanin and infection, if present, appears weak in *D. tinctorius*, possibly due to low variation in melanin allocation across individuals, or because other traits are more important mediators of *Bd* susceptibility in this species.

### Carotenoid-based coloration

Our findings did not support the hypothesis that the brightness, saturation or hue of the dorsal yellow coloration reflects *Bd* infection status, contributing to a growing body of literature reporting inconsistent links between parasite load and carotenoid-based coloration in amphibians as opposed to other taxa (Cothran et al. 2015; Prokopius et al. 2025). While extensive evidence from birds and fish supports the role of carotenoid-based ornaments as honest signals of health and immune status in the context of sexual selection (eg, Milinski and Bakker 1990; Houde and Torio 1992; Hill and Brawner 1998; Penha et al. 2020), such associations are far less consistent in amphibians. For example, Longo et al. (2020) found that ticks preferentially parasitized males of Puerto Rican rock frogs (*Eleutherodactylus cooki*) with a larger extent of yellow coloration, while no effect was found for *Bd* infection. Likewise, Auld (2024) reported no *Bd*-related effect on the yellow shoulder patches of Brown toadlet (*Pseudophryne bibronii*) males, and Pröhl et al. (2013) found only a weak influence of nematode infection in strawberry poison frog (*Oophaga pumilio*) visual contrast.

The absence of a color-infection relationship in our study may reflect low overall infection severity. Body condition did not predict the probability or loads of *Bd* infection in *D. tinctorius*, implying lower physiological stress compared with other species. This could indicate that individuals in poor condition are not more susceptible to infection, or alternatively, that the observed intensities of *Bd* infection do not impair feeding behavior or increase metabolic costs in a way that would affect dietary carotenoid intake and the resulting coloration (Peterson et al. 2013; McQuigg et al. 2023). A recent experimental study in *Oophaga pumilio* found that even high *Bd* infection loads had no effect on skin carotenoid concentrations when frogs were supplemented with dietary carotenoids, suggesting that carotenoid-based pigmentation may be maintained despite infection (Prokopius et al. 2025). Such results suggest that infection alone may not impair coloration unless it imposes substantial energetic constraints.

Instead, we found that body size (SVL) influenced the probability and intensity of infection, as well as coloration. In particular, saturation of the yellow patches increased with SVL in females but decreased in males. If we consider body size as a proxy for age, the observed patterns may reflect ontogenetic shifts in pigmentation and/or immune investment. These patterns may reflect differing life history strategies or energy allocation trade-offs between sexes, which could contribute to the observed differences in infection dynamics between males and females (Longo and Burrowes 2010; Langhammer et al. 2014). However, to clarify these complex relationships, we would need a broader range of body sizes and a larger sample size.

The lack of associations between *Bd* infection and either melanin- or carotenoid-based coloration may reflect the complexity of factors influencing pigmentation and disease dynamics in wild populations. Environmental heterogeneity, genetic variation in color or immune traits, or context-dependent trade-offs could obscure subtle relationships. Additionally, dorsal coloration in *D. tinctorius* likely functions primarily as an aposematic signal to predators rather than as a condition-dependent cue in intraspecific communication. If so, aposematic signals may still constitute costly but evolutionary stable traits, showing limited sensitivity to short-term physiological variation under enzootic infection conditions. It remains to be investigated if coloration in other body parts, potentially more important in social signaling such as the vocal sac or frontal area, show greater sensitivity to parasite infection and condition.

## Conclusions

Our study contributes to a growing body of evidence showing that relationships between coloration and individual quality, such as infection status, may be less universal and more context- and taxa-dependent than often assumed. While such links are well-established in birds and fish, our findings show that dorsal melanin- and carotenoid-based coloration in *Dendrobates tinctorius* do not appear to reflect infection status or intensity in wild populations. This suggests that, at least under current infection conditions in the focal species, external coloration may not serve as a reliable indicator of infection status and load. Instead, pigmentation traits in open populations may be influenced by a combination of ecological, developmental, and evolutionary factors, including their primary role in anti-predator signaling. Further research is needed to understand when and how coloration reflects individual condition in amphibians, particularly in species with high phenotypic diversity and exposure to emerging pathogens.

## Supplementary Material

araf137_Supplementary_Data

## Data Availability

Analyses reported in this article can be reproduced using the data and R script provided by Schlippe Justicia et al. (2025).
