# Supplementary material for: Defensive coloration is not a reliable indicator of fungal infection in aposematic poison frogs
Source: Behav Ecol. 2025 Nov 20;37(1):araf137. doi: 10.1093/beheco/araf137 (PMC12705270; doi:10.1093/beheco/araf137)
Supplement: araf137_Supplementary_Data [file araf137_supplementary_data.docx]

**SUPPLEMENTARY MATERIAL**

*Defensive colouration is not a reliable indicator of fungal infection in aposematic poison frogs*

*Lia Schlippe Justicia, Carolin Dittrich, Ossi Nokelainen, Bibiana Rojas*

**Supplementary material S1 - Primer and Probe sequences for ddPCR**

*Batrachochytrium dendrobatidis (Bd)*

Primers used for detection of *Bd:* ITS 1-3 and S5.8

ITS-1 reverse primer: CCTTGATATAATACAGTGTGCCATATGTC

rDNA S5.8 forward primer: CGA GTC GAA CAA AAT

FAM labelled dye targets the minor binding groove (after Boyle et al. 2004): [FAM] CGA GTC GAA CAA AAT [MGBEQ]

*Dendrobates tinctorius*

RAG1 gene as amplification control for ddPCR

RAG1 - TG1R tinct reverse primer: CTGAACAGTTTATTACCGGACTCG

RAG1 - TG1F tinct forward primer: CCAGCTGGAAATAGGAGAAGTCTA

HEX labelled probe for RAG1 gene: [HEX] TTGCCGAACTCTTGTCCACCAAG [BHQ1]

**Supplementary material S2 – Full models**

**Table S2a.** Full model estimate, standard error (SE), lower (LCI) and upper (UCI) 95% confidence intervals for the analysis of (a) probability of *Bd* infection and (b) *Bd* load for *Dendrobates tinctorius* adults from five populations (n = 212 adults). Informative parameters are shown in bold (95% confidence intervals do not overlap zero).

| **Parameter** | **Estimate** | **SE** | **LCI** | **UCI** |
| --- | --- | --- | --- | --- |
| *(a) Bd infection status* |  |  |  |  |
| **Intercept** | **-0.78** | **0.34** | **-1.55** | **-0.04** |
| Sex (Male) | -0.71 | 0.40 | -1.52 | 0.07 |
| SMI | -0.12 | 0.19 | -0.51 | 0.25 |
| **SVL** | **-1.13** | **0.42** | **-2.01** | **-0.35** |
| **Sex (Male)** × **SVL** | **1.54** | **0.76** | **0.09** | **3.10** |
| *(b) Bd load* |  |  |  |  |
| Intercept | 1.82 | 1.04 | -0.28 | 3.92 |
| Sex (Male) | -0.52 | 0.86 | -2.27 | 1.24 |
| SMI | -0.61 | 0.34 | -1.30 | 0.07 |
| **SVL** | **-2.09** | **0.73** | **-3.57** | **-0.62** |
| Population (Mataroni) | 0.05 | 1.36 | -2.69 | 2.80 |
| Population (Mt. Fortuné) | 0.33 | 1.18 | -2.07 | 2.72 |
| Population (Nouragues) | 0.99 | 1.42 | -1.88 | 3.87 |
| Population (P. Matoury) | 2.15 | 1.34 | -0.57 | 4.86 |
| Sex (Male) × SVL | 0.24 | 1.60 | -2.99 | 3.48 |

**Table S2b**. Full model estimates ± standard errors (SE), and 95% confidence intervals (LCI, UCI) for the analysis of **brightness** of the dorsal **black colouration** of *Dendrobates tinctorius*. Results are shown for models with the explanatory variable *Bd* representing (a) ***Bd* infection status** (“Yes” corresponds to intercept level) and (b) ***Bd* load**. Informative parameters are shown in bold (95% confidence intervals do not overlap zero).

|  | *(a) Bd infection status* | | *(a) Bd load* | | |
| --- | --- | --- | --- | --- | --- |
| **Parameter** | **Estimate ± SE** | **95% CI** | | **Estimate ± SE** | **95% CI** |
| **Intercept** | **0.34 ± 0.02** | **0.30, 0.38** | | **0.37 ± 0.04** | **0.28, 0.46** |
| Sex (Male) | -0.01 **±** 0.02 | -0.05, 0.02 | | 0.05 **±** 0.03 | -0.02, 0.12 |
| *Bd* | 0.03 **±** 0.05 | -0.06, 0.12 | | -0.02 **±** 0.02 | -0.05, 0.01 |
| SMI | -0.01 **±** 0.01 | -0.03, 0.01 | | 0.03 **±** 0.02 | -0.02, 0.08 |
| SVL | 0.02 **±** 0.02 | -0.01, 0.05 | | 0.03 ± 0.04 | -0.04, 0.11 |
| **Population (Mataroni)** | **-0.06 ± 0.02** | **-0.11, -0.01** | | -0.09 **±** 0.06 | -0.22, 0.04 |
| Population (Mt. Fortuné) | 0.02 **±** 0.03 | -0.03, 0.08 | | -0.02 **±** 0.05 | -0.13, 0.09 |
| Population (Nouragues) | -0.04 **±** 0.02 | -0.08, 0.01 | | -0.02 ± 0.07 | -0.16, 0.12 |
| Population (P. Matoury) | 0.03 **±** 0.03 | -0.03, 0.08 | | < -0.01 ± 0.07 | -0.13, 0.13 |
| **Sex (Male)** × **SVL** | **-0.07 ± 0.03** | **-0.13, -0.01** | | -0.12 ± 0.06 | -0.25, 0.01 |
| *Bd* × Population (Mataroni) | -0.03 **±** 0.06 | -0.15, 0.09 | | 0.01 ± 0.02 | -0.03, 0.06 |
| *Bd* × Population (Mt. Fortuné) | < -0.01 **±** 0.06 | -0.11, 0.11 | | 0.03 ± 0.02 | -0.01, 0.07 |
| *Bd* × Population (Nouragues) | 0.08 **±** 0.06 | -0.05, 0.20 | | 0.03 **±** 0.02 | -0.02, 0.08 |
| *Bd* × Population (P. Matoury) | -0.02 **±** 0.06 | -0.14, 0.10 | | 0.02 **±** 0.02 | -0.02, 0.06 |
| *Bd* × SMI | 0.02 **±** 0.02 | -0.01, 0.06 | | < -0.01 ± 0.01 | -0.01, 0.01 |

**Table S2c**. Full model estimates ± standard errors (SE), and 95% confidence intervals (LCI, UCI) for the analysis of **brightness** of the dorsal **yellow colouration** of *Dendrobates tinctorius*. Results are shown for models with the explanatory variable *Bd* representing (a) ***Bd* infection status** (“Yes” corresponds to intercept level) and (b) ***Bd* load**. Informative parameters are shown in bold (95% confidence intervals do not overlap zero).

|  | *(a) Bd infection status* | | *(a) Bd load* | | |
| --- | --- | --- | --- | --- | --- |
| **Parameter** | **Estimate ± SE** | **95% CI** | | **Estimate ± SE** | **95% CI** |
| **Intercept** | **3.65 ±** **0.08** | **3.50, 3.80** | | **3.81 ± 0.16** | **3.47, 4.14** |
| Sex (Male) | 0.02 ± 0.06 | -0.11, 0.14 | | 0.19 ± 0.13 | -0.06, 0.45 |
| *Bd* | 0.01 ± 0.16 | -0.32, 0.33 | | **-0.15 ± 0.06** | **-0.27, -0.03** |
| SMI | 0.02 ± 0.04 | -0.05, 0.09 | | -0.02 ± 0.08 | -0.19, 0.14 |
| SVL | 0.07 ± 0.06 | -0.05, 0.20 | | -0.10 ± 0.13 | -0.36, 0.16 |
| Population (Mataroni) | -0.12 ± 0.09 | -0.29, 0.06 | | -0.22 ± 0.22 | -0.67, 0.22 |
| **Population (Mt. Fortuné)** | **0.44 ± 0.11** | **0.23, 0.66** | | 0.35 ± 0.19 | -0.04, 0.75 |
| Population (Nouragues) | -0.04 ± 0.09 | -0.22, 0.14 | | -0.28 ± 0.25 | -0.79, 0.23 |
| **Population (P. Matoury)** | **0.43 ± 0.10** | **0.23, 0.62** | | **0.50 ± 0.24** | **0.01, 0.99** |
| Sex (Male) × SVL | -0.15 ± 0.11 | -0.37, 0.07 | | 0.10 ± 0.23 | -0.37, 0.57 |
| *Bd* × Population (Mataroni) | 0.02 ± 0.22 | -0.42, 0.45 | | 0.13 ± 0.08 | -0.03, 0.30 |
| *Bd* × Population (Mt. Fortuné) | 0.04 ± 0.21 | -0.37, 0.45 | | 0.13 ± 0.07 | -0.02, 0.28 |
| *Bd* × Population (Nouragues) | 0.04 ± 0.23 | -0.41, 0.49 | | 0.15 ± 0.08 | -0.02, 0.32 |
| *Bd* × Population (P. Matoury) | -0.02 ± 0.22 | -0.46, 0.42 | | 0.07 ± 0.07 | -0.07, 0.22 |
| *Bd* × SMI | -0.07 ± 0.06 | -0.19, 0.05 | | -0.01 ± 0.02 | -0.05, 0.04 |

**Table S2d**. Full model estimates ± standard errors (SE), and 95% confidence intervals (LCI, UCI) for the analysis of **saturation** of the dorsal **yellow colouration** of *Dendrobates tinctorius*. Results are shown for models with the explanatory variable *Bd* representing (a) ***Bd* infection status** (“Yes” corresponds to intercept level) and (b) ***Bd* load**. Informative parameters are shown in bold (95% confidence intervals do not overlap zero).

|  | *(a) Bd infection status* | | | *(a) Bd load* | |
| --- | --- | --- | --- | --- | --- |
| **Parameter** | **Estimate ± SE** | **95% CI** | **Estimate ± SE** | | **95% CI** |
| **Intercept** | **3.72 ± 0.08** | **3.57, 3.88** | **3.79 ± 0.21** | | **3.37, 4.21** |
| Sex (Male) | 0.04 ± 0.06 | -0.09, 0.17 | 0.24 ± 0.16 | | -0.09, 0.56 |
| *Bd* | -0.01 ± 0.17 | -0.34, 0.32 | -0.13 ± 0.08 | | -0.29, 0.02 |
| SMI | 0.04 ± 0.04 | -0.03, 0.12 | -0.08 ± 0.10 | | -0.30, 0.13 |
| **SVL** | **0.17 ± 0.06** | **0.05, 0.29** | 0.05 ± 0.16 | | -0.28, 0.38 |
| Population (Mataroni) | 0.10 ± 0.09 | -0.08, 0.28 | 0.14 ± 0.28 | | -0.43, 0.70 |
| **Population (Mt. Fortuné)** | **-1.04 ± 0.11** | **-1.25, -0.83** | **-1.12 ± 0.24** | | **-1.62, -0.62** |
| Population (Nouragues) | 0.07 ± 0.09 | -0.11, 0.25 | -0.08 ± 0.32 | | -0.73, 0.57 |
| **Population (P. Matoury)** | **-1.19 ± 0.10** | **-1.39, -0.99** | **-1.33 ± 0.30** | | **-1.94, -0.71** |
| **Sex (Male) × SVL** | **-0.26 ± 0.11** | **-0.48, -0.04** | -0.28 ± 0.29 | | -0.88, 0.32 |
| *Bd* × Population (Mataroni) | 0.10 ± 0.22 | -0.33, 0.54 | 0.14 ± 0.10 | | -0.07, 0.35 |
| *Bd* × Population (Mt. Fortuné) | 0.12 ± 0.21 | -0.30, 0.53 | **0.20 ± 0.09** | | **0.02, 0.39** |
| *Bd* × Population (Nouragues) | 0.08 ± 0.23 | -0.37, 0.54 | 0.14 ± 0.11 | | -0.07, 0.36 |
| *Bd* × Population (P. Matoury) | 0.04 ± 0.23 | -0.40, 0.49 | 0.15 ± 0.09 | | -0.04, 0.33 |
| *Bd* × SMI | -0.08 ± 0.06 | -0.20, 0.04 | 0.03 ± 0.03 | | -0.02, 0.08 |

**Supplementary material S3 – Model selection**

**Table S3.** Overview of the model selection for the analysis of **brightness** of the dorsal **black** colouration of *Dendrobates tinctorius,* including models with (a) ***Bd* infection status** (*Bd*^+^ vs *Bd*^-^) and (b) ***Bd* load** as predictors. Models are ranked by AIC.

| **Model** | **Variables** | **df** | **logLik** | **AICc** | **delta AIC** | **AIC weight** |
| --- | --- | --- | --- | --- | --- | --- |
| *(a) Bd infection status* | | | | | | |
| **Best** | Population | 6 | 177.62 | -342.8 | 0.00 | 0.99 |
| **Intercept** | Intercept only | 2 | 168.71 | -333.4 | 9.46 | 0.01 |
| **Full** | *Bd* infection + Sex + Population + SMI + SVL + SVL×Sex +  *Bd* infection×Population +  *Bd* infection×SMI | 16 | 183.69 | -332.4 | 10.38 | <0.01 |
| *(b) Bd load* | |  |  |  |  |  |
| **Best** | Sex + SMI | 4 | 44.51 | -80.0 | 0.00 | 0.55 |
| **Intercept** | Intercept only | 2 | 41.93 | -79.6 | 0.42 | 0.45 |
| **Full** | Sex + *Bd* load + SMI + SVL + SVL×Sex + *Bd* load×Population + *Bd* load×SMI | 16 | 51.42 | -50.7 | 29.29 | 0.00 |

**Supplementary material S4 - Populations pairwise comparisons**

**Table S4a.** Post hoc pairwise comparison (Tukey-adjusted) of the **brightness** values of *Dendrobates tinctorius’* dorsal **black colouration** among five populations. The table includes the estimated difference in brightness between population pairs, standard errors (SE), 95% confidence intervals (CI), t-ratios, and adjusted p-values. Statistically significant differences (p < 0.05) are highlighted in bold.

| **Pairwise populations** | **Estimate** | **SE** | **95% CI** | **t-ratio** | **p-value (Tukey)** |
| --- | --- | --- | --- | --- | --- |
| Kaw - Mataroni | 0.05 | 0.02 | [-0.01, 0.11] | 2.39 | 0.12 |
| Kaw - Mont Fortuné | -0.03 | 0.02 | [-0.10, 0.03] | -1.44 | 0.60 |
| Kaw - Nouragues | 0.01 | 0.02 | [-0.05, 0.07] | 0.57 | 0.98 |
| Kaw - Petit Matoury | -0.02 | 0.02 | [-0.09, 0.05] | -0.82 | 0.93 |
| **Mataroni - Mont Fortuné** | **-0.09** | **0.02** | **[-0.15, -0.03]** | **-3.87** | **<0.01** |
| Mataroni - Nouragues | -0.04 | 0.02 | [-0.10, 0.02] | -1.96 | 0.29 |
| **Mataroni - Petit Matoury** | **-0.07** | **0.02** | **[-0.14, -0.01]** | **-3.17** | **0.02** |
| Mont Fortuné - Nouragues | 0.05 | 0.02 | [-0.01, 0.11] | 2.09 | 0.23 |
| Mont Fortuné - Petit Matoury | 0.02 | 0.02 | [-0.05, 0.08] | 0.59 | 0.98 |
| Nouragues - Petit Matoury | -0.03 | 0.02 | [-0.10, 0.03] | -1.42 | 0.62 |

**Table S4b**. Post hoc pairwise comparison (Tukey-adjusted) of the **brightness** values of *Dendrobates tinctorius’* dorsal **yellow colouration** among five populations. The table includes the estimated difference in brightness between population pairs, standard errors (SE), 95% confidence intervals (CI), t-ratios, and adjusted p-values. Statistically significant differences (p < 0.05) are highlighted in bold.

| **Pairwise populations** | **Estimate** | **SE** | **95% CI** | **t-ratio** | **p-value (Tukey)** |
| --- | --- | --- | --- | --- | --- |
| Kaw - Mataroni | 0.11 | 0.08 | [-0.11, 0.33] | 1.37 | 0.65 |
| **Kaw - Mont Fortuné** | **-0.47** | **0.09** | **[-0.71, -0.24]** | **-5.50** | **<0.01** |
| Kaw - Nouragues | 0.02 | 0.08 | [-0.20, 0.24] | 0.26 | 1.00 |
| **Kaw - Petit Matoury** | **-0.40** | **0.09** | **[-0.64, -0.17]** | **-4.66** | **<0.01** |
| **Mataroni - Mont Fortuné** | **-0.58** | **0.08** | **[-0.80, -0.36]** | **-7.16** | **<0.01** |
| Mataroni - Nouragues | -0.09 | 0.07 | [-0.29, 0.12] | -1.20 | 0.75 |
| **Mataroni - Petit Matoury** | **-0.51** | **0.08** | **[-0.74, -0.29]** | **-6.25** | **<0.01** |
| **Mont Fortuné - Nouragues** | **0.49** | **0.08** | **[0.27, 0.71]** | **6.12** | **<0.01** |
| Mont Fortuné - Petit Matoury | 0.07 | 0.09 | [-0.18, 0.31] | 0.75 | 0.95 |
| **Nouragues - Petit Matoury** | **-0.42** | **0.08** | **[-0.65, -0.20]** | **-5.21** | **<0.01** |

**Table S4c.** Post hoc pairwise comparison (Tukey-adjusted) of the **saturation** values of *Dendrobates tinctorius’* dorsal **yellow colouration** among five populations. The table includes the estimated difference in brightness between population pairs, standard errors (SE), 95% confidence intervals (CI), t-ratios, and adjusted p-values. Statistically significant differences (p < 0.05) are highlighted in bold.

| **Pairwise populations** | **Estimate** | **SE** | **95% CI** | **t-ratio** | **p-value (Tukey)** |
| --- | --- | --- | --- | --- | --- |
| Kaw - Mataroni | -0.10 | 0.08 | [-0.33, 0.12] | -1.27 | 0.71 |
| **Kaw - Mont Fortuné** | **0.99** | **0.09** | **[0.75, 1.23]** | **11.35** | **<0.01** |
| Kaw - Nouragues | -0.08 | 0.08 | [-0.31, 0.15] | -0.97 | 0.87 |
| **Kaw - Petit Matoury** | **1.18** | **0.09** | **[0.94, 1.43]** | **13.32** | **<0.01** |
| **Mataroni - Mont Fortuné** | **1.09** | **0.08** | **[0.87, 1.32]** | **13.30** | **<0.01** |
| Mataroni - Nouragues | 0.02 | 0.08 | [-0.18, 0.23] | 0.33 | 0.10 |
| **Mataroni - Petit Matoury** | **1.29** | **0.09** | **[1.05, 1.53]** | **14.90** | **<0.01** |
| **Mont Fortuné - Nouragues** | **-1.07** | **0.08** | **[-1.30, -0.84]** | **-12.96** | **<0.01** |
| Mont Fortuné - Petit Matoury | 0.19 | 0.09 | [-0.06, 0.44] | 2.14 | 0.21 |
| **Nouragues - Petit Matoury** | **1.26** | **0.09** | **[1.02, 1.51]** | **14.39** | **<0.01** |

**Table S4d.** Pairwise comparisons of estimated mean **hue** (in degrees) of the dorsal **yellow** **colouration** in *Dendrobates tinctorius* across five populations, based on posterior distributions from a circular regression model. Shown are the mean differences between population pairs, along with the lower (LB) and upper (UB) bounds of the 95% credible intervals. Values whose credible intervals do not overlap 0 are shown in bold.

| **Pairwise populations** | **Mean Difference** | **LB** | **UB** |
| --- | --- | --- | --- |
| **Kaw - Mataroni** | **14.30** | **11.97** | **16.65** |
| **Kaw - Mont Fortuné** | **-58.43** | **-69.61** | **-48.32** |
| **Kaw - Nouragues** | **9.28** | **6.75** | **11. 84** |
| **Kaw - Petit Matoury** | **-52.18** | **-63.94** | **-41.80** |
| **Mataroni - Mont Fortuné** | **-72.73** | **-84.02** | **-62.53** |
| **Mataroni - Nouragues** | **-5.02** | **-6.95** | **-3.14** |
| **Mataroni - Petit Matoury** | **-66.48** | **-78.32** | **-55.97** |
| **Mont Fortuné - Nouragues** | **67.70** | **57.51** | **78.97** |
| Mont Fortuné - Petit Matoury | 6.25 | –7.03 | 19.32 |
| **Nouragues - Petit Matoury** | **-61.45** | **-73.22** | **-50.95** |

***(Not referred in the main text)***

**Additional supplementary material S5 & S6 - Model selection**

**Table S5.** Overview of the model selection for the analysis of (a) probability of *Bd* infection and (b) *Bd* load for *Dendrobates tinctorius* adults from five populations (n = 212 adults). Models are ranked by AIC.

| **Model** | **Variables** | **df** | **logLik** | **AICc** | **delta AIC** | **AIC weight** |
| --- | --- | --- | --- | --- | --- | --- |
| *(a) Bd prevalence* | | | | | | |
| **Best** | Sex + SVL + SVL×Sex | 5 | -104.11 | 218.5 | 0.00 | 0.62 |
| **Full** | Sex + SMI + SVL + SVL×Sex | 6 | -103.91 | 220.2 | 1.71 | 0.26 |
| **Intercept** | Intercept only | 2 | -108.91 | 221.9 | 3.37 | 0.12 |
| *(b) Bd load* |  |  |  |  |  |  |
| **Best** | SMI + SVL | 4 | -105.87 | 220.7 | 0.00 | 0.98 |
| **Intercept** | Intercept only | 2 | -112.23 | 228.7 | 6.66 | 0.04 |
| **Full** | Sex + SMI + SVL + Population + SVL×Sex | 10 | -103.44 | 233.2 | 12.44 | <0.01 |

**Table S6a.** Overview of the model selection for the analysis of **brightness** of the dorsal **yellow** colouration of *Dendrobates tinctorius,* including models with (a) ***Bd* infection status** (*Bd*^+^ vs *Bd*^-^) and (b) ***Bd* load** as predictors. Models are ranked by AIC.

| **Model** | **Variables** | **df** | **logLik** | **AICc** | **delta AIC** | **AIC weight** |
| --- | --- | --- | --- | --- | --- | --- |
| *(a) Bd infection status* | | | | | | |
| **Best** | Population | 6 | -78.43 | 169.3 | 0.00 | 1.00 |
| **Full** | *Bd* infection + Sex + Population + SMI + SVL + SVL×Sex +  *Bd* infection×Population +  *Bd* infection×SMI | 16 | -76.48 | 187.9 | 18.62 | 0.00 |
| **Intercept** | Intercept only | 2 | -114.02 | 232.1 | 62.81 | 0.00 |
| *(b) Bd load* |  |  |  |  |  |  |
| **Best** | Population + *Bd* load | 7 | -13.26 | 43.6 | 0.00 | 0.997 |
| **Intercept** | Intercept only | 2 | -25.62 | 55.5 | 11.97 | 0.003 |
| **Full** | *Bd* load + Sex + SMI + SVL + Population + SVL×Sex + *Bd* load×Population + *Bd* load×SMI | 16 | -5.60 | 62.6 | 19.07 | 0.00 |

**Table S6b.** Overview of the model selection for the analysis of **saturation** of the dorsal **yellow** colouration of *Dendrobates tinctorius,* including models with (a) ***Bd* infection status** (*Bd*^+^ vs *Bd*^-^) and (b) ***Bd* load** as predictors. Models are ranked by AIC.

| **Model** | **Variables** | **df** | **logLik** | **AICc** | **delta AIC** | **AIC weight** |
| --- | --- | --- | --- | --- | --- | --- |
| *(a) Bd infection status* | | | | | | |
| **Best** | Sex + Population + SVL + SVL×Sex | 9 | -79.96 | 178.9 | 0.00 | 0.998 |
| **Full** | *Bd* infection + Sex + Population + SMI + SVL + SVL×Sex +  *Bd* infection×Population +  *Bd* infection×SMI | 16 | -78.07 | 191.1 | 12.25 | 0.002 |
| **Intercept** | Intercept only | 2 | -205.07 | 414.2 | 235.35 | 0.00 |
| *(b) Bd load* | | | | | | |
| **Best** | Sex + Population | 7 | -20.84 | 58.7 | 0.00 | 1.00 |
| **Intercept** | Intercept only | 16 | -16.32 | 84.1 | 25.36 | 0.00 |
| **Full** | *Bd* load + Sex + SMI + SVL + Population + SVL×Sex + *Bd* load×Population | 2 | -47.15 | 98.6 | 39.89 | 0.00 |

**Additional supplementary material S7 - Models summary**

**Table S7.** Estimate, standard error (SE), lower (LCI) and upper (UCI) 95% confidence intervals for the analysis of dorsal **black colouration** in 212 *Dendrobates tinctorius* from five populations, including (a) ***Bd* infection status** and (b) ***Bd* load** as explanatory variables. Informative parameters are shown in bold (95% confidence intervals do not overlap zero).

| **Parameter** | **Estimate** | **SE** | **LCI** | **UCI** |
| --- | --- | --- | --- | --- |
| *(a) Bd infection status* |  |  |  |  |
| **Intercept** | **0.35** | **0.02** | **0.31** | **0.38** |
| **Population (Mataroni)** | **-0.05** | **0.02** | **-0.10** | **-0.01** |
| Population (Mt. Fortuné) | 0.03 | 0.02 | -0.01 | 0.08 |
| Population (Nouragues) | -0.01 | 0.02 | -0.06 | 0.03 |
| Population (P. Matoury) | 0.02 | 0.02 | -0.03 | 0.07 |
| *(b) Bd load* |  |  |  |  |
| **Intercept** | **0.35** | **0.02** | **0.32** | **0.39** |
| Sex (Male) | 0.05 | 0.03 | -0.01 | 0.11 |
| SMI | 0.02 | 0.01 | -0.01 | 0.05 |
